# Nicotinamide: Bright Potential in Glaucoma Management

**DOI:** 10.3390/biomedicines12081655

**Published:** 2024-07-25

**Authors:** Silvia Babighian, Irene Gattazzo, Maria Sole Zanella, Alessandro Galan, Fabiana D’Esposito, Mutali Musa, Caterina Gagliano, Lucia Lapenna, Marco Zeppieri

**Affiliations:** 1 Department of Ophthalmology, Ospedale Sant’Antonio, Azienda Ospedaliera, 35127 Padova, Italy; silvia.babighian@aopd.veneto.it (S.B.);; 2 Imperial College Ophthalmic Research Group (ICORG) Unit, Imperial College, 153-173 Marylebone Rd, London NW15QH, UK; 3 Department of Neurosciences, Reproductive Sciences and Dentistry, University of Naples Federico II, Via Pansini 5, 80131 Napoli, Italy; 4 Department of Optometry, University of Benin, Benin City 300238, Nigeria; 5 Africa Eye Laser Centre, Km 7, Benin 300105, Nigeria; 6 Department of Medicine and Surgery, University of Enna “Kore”, Piazza dell’Università, 94100 Enna, Italy; 7 Eye Clinic Catania University San Marco Hospital, Viale Carlo Azeglio Ciampi, 95121 Catania, Italy; 8 U.O.C Oculistica, Ospedale “DI Venere”, 70012 Bari, Italy; 9 Department of Ophthalmology, University Hospital of Udine, 33100 Udine, Italy

**Keywords:** nicotinamide, glaucoma, intraocular pressure

## Abstract

Background: Glaucoma is a major cause of incurable ocular morbidity and poses significant challenges in its management due to the limited treatment options and potential adverse effects. Nicotinamide, a naturally occurring diet-rich nutrient, has emerged as a promising therapeutic agent for glaucoma, offering neuroprotective effects and the potential modulation of intraocular pressure (IOP) regulation pathways. This comprehensive review sought to analyze the current literature on nicotinamide in glaucoma management, exploring its mechanisms of action, efficacy, and safety profile. Methods: A systematic search of the PubMed database was conducted to identify relevant records on the therapeutic actions of nicotinamide in ocular hypertension and glaucoma. Publications evaluating nicotinamide’s effects on retinal ganglion cells (RGCs), optic nerve function, IOP regulation, and neuroinflammatory pathways were included. Results: The literature review revealed the preclinical evidence supporting nicotinamide’s neuroprotective effects on RGCs, the preservation of optic nerve integrity, and the modulation of glaucoma-associated neuroinflammation. Additionally, nicotinamide may exert IOP-lowering effects through its influence on ocular blood flow and aqueous humor dynamics. Conclusions: Nicotinamide holds promise as a novel therapeutic approach in glaucoma management, offering potential neuroprotective and IOP-lowering effects. The authors recommend more research to determine the nicotinamide efficacy, safe dosing parameters, and any long-term safety concerns in glaucoma patients.

## 1. Introduction

Glaucomas are a diverse group of conditions that cause irreversible vision loss and affect over 70 million people all over the world [1]. The hallmark of glaucoma is a gradual destruction of retinal ganglion cells (RGCs) and optic nerve injury, with abnormal intraocular pressure (IOP) being the primary cause [2]. As a result, glaucoma management requires the administration of hypotensive pharmacological agents to reduce IOP or surgery [3]. 

Glaucoma presents as either open-angle, or closed-angle. Primary open-angle glaucoma presents with an anterior chamber (AC) angle which is open/deep, suggesting that the aqueous humor outflow is not impeded. In closed-angle glaucoma, the AC is narrow, or even closed, suggesting that the aqueous humor outflow is actively impeded [1]. Glaucoma can be classified as either primary or secondary, depending on whether it develops independently (primary) or as a consequence of another disease process. Given that glaucoma often presents without noticeable symptoms in its initial stages, approximately 10–50% of those affected are typically diagnosed only at advanced stages of the disease. Consequently, a search for an effective therapeutic approach to glaucoma remains a top priority [4]. Currently, any vision loss caused by glaucoma cannot be retrieved by any known means. Therefore, early detection and effective treatment are crucial. Due to the emergence of better healthcare options to society, people are generally expected to live longer, and this will come with a higher incidence and prevalence of glaucoma, highlighting the need for improved detection methods and quality care to prevent unnecessary blindness [5]. 

The first line of management for glaucoma is prostaglandin analogues, closely followed by beta-blockers, alpha-agonists, etc. [4]. All these therapies are currently aimed at reducing intraocular pressure, but do little in terms of protecting the neural tissue potentially damaged by the disease [4]. 

Nowadays, the goal of glaucoma research is to find intraocular-pressure-independent techniques to reducing the likelihood and severity of glaucomatous optic nerve damage. Recent advances in trial design and technology developments that allow for the early detection of pathogenic alterations have revived interest in neuroprotection research. This increased interest is fueled by a clearer knowledge of the processes behind RGC degeneration and breakthroughs in basic research targeted at finding possible treatment targets [5].

While a definitive consensus on the precise cause of glaucomatous optic neuropathy remains elusive, the cellular mechanisms leading to RGC death encompass the exposure to neurotoxic substances like nitric oxide (NO) and glutamate, as well as the deprivation of internal trophic factors, impairment of cellular self-repair processes, and intracellular destructive pathways [6].

The rationale behind the treatment lies in restoring the balance between cellular death and survival signals, thereby preserving visual function. Consequently, numerous ongoing trials are investigating the potential benefits of neuroprotective agents including Memantine, Brimonidine, nerve growth factor (NGF), and Nicotinamide [7]. Despite the limited clinical validation confirming its therapeutic properties in glaucoma management, Nicotinamide (Vitamin B3) is already advocated as an adjunct treatment for this disease especially in sufferers experiencing the progression of visual field loss despite adequate intraocular pressure control [8,9].

**Figure 1:**
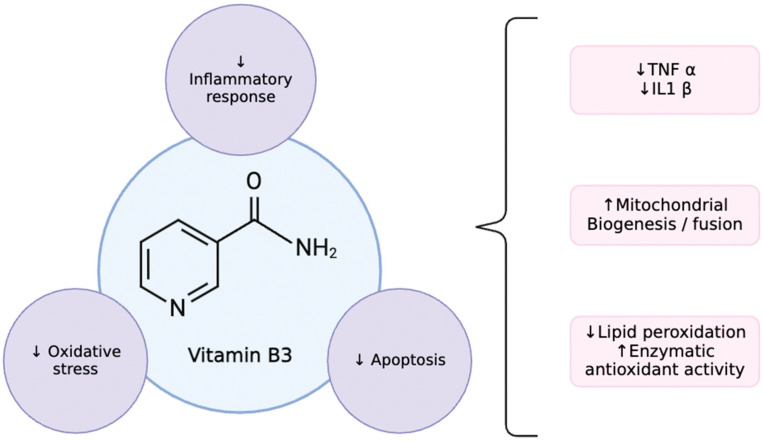
NAD structure and mechanisms of action. Up arrow = increase, downward arrow = decrease.

Nicotinamide is a water-soluble nutrient which is largely obtained by consuming nutrient-rich foods such as poultry and fish [7]. Its chemical structure is shown below in Figure 1.

## 2. Materials and Methods

For this review, an electronic search was carried out using PubMed and Medline databases for all published articles up to 20 May 2024. The comprehensive literature analysis included articles published between 2000 and 2024, utilizing a combination of the following terms: nicotinamide, glaucoma, intraocular pressure, nicotinamide riboside, and neuroprotection. A total of five results were returned. Relevant cross-references from the selected articles were also included in the review. Records which were not in English language or out of scope as related to this manuscript were excluded. Moreover, records without complete research data and those with errata were excluded. The literature search was performed by three authors (I.G., M.Z., and S.B.). Any disagreements were resolved through open discussion with senior authors.

## 3. Nicotinamide: Mechanisms of Action

Nicotinamide (NAM) is a substance that is water-soluble. It is part of the niacin and/or vitamin B3 complex, together with nicotinic acid (NA) and nicotinamide riboside (NR). It acts as a precursor to nicotinamide adenine dinucleotide (NAD+), and is crucial for essential cellular activities [10]. These substances are precursors in the food for the production of the beneficial molecules nicotinamide adenine dinucleotide without (NAD) and with phosphate (NADP). Nicotinamide is an essential part of the glycolysis pathway. It helps in the production of NAD+ which is necessary for generating ATP and controlling cellular energy levels as well as several metabolic activities [11].

There are three types of vitamin B3, of which the most commonly encountered as a component of human diet are NAM and NA. Upon intestinal absorption in the human body, NA is converted into NAM [12]. A lesser proportion of NAM is retained in the liver, as the majority is either removed as urine or utilized in the production of critical substances such as NAD [13,14,15]. NAM is acquired through dietary sources, predominantly found in eggs, beef, fish, and mushrooms, with lesser quantities present in vegetables. Additionally, nicotinamide is derived from dietary tryptophan. Tryptophan is an essential amino acid [16].

The properties attributed to NAM are manifold, encompassing anti-inflammatory, neuroprotective, and antioxidant effects. Concerning its anti-inflammatory properties, it has photoprotective properties on the skin, reducing hyperpigmentation, wrinkles, ultraviolet-exposure-related immunosuppression, and sebum synthesis [12]. Consequently, it finds application in dermatology for the treatment of conditions such as pemphigoid, acne, skin cancer, and atopic dermatitis [17], as well as in rheumatology as an antipsoriatic agent [18]. 

Nicotinamide’s neuroprotective impact is due to its role in neuronal formation, survival, and function in the central nervous system. Its function in the production of NAD emphasizes its critical position for cells prone to diminishing NAD amounts, notably neuronal cells [13], which have been shown to decline with age [19]. As a result, nicotinamide is expected to be of critical importance in neuronal development and neuroprotection, contributing to both neuronal death and neuroprotective activity. Numerous studies emphasize its role in neurological diseases and neurodegenerative illnesses [20]. 

Furthermore, NAM has antioxidant capabilities that maintain membrane integrity, and prevent cellular damage, phagocytosis, programmed cell death, and the development of venous thrombosis [21]. Furthermore, it appears to be implicated as a treatment factor in three major neurodegenerative conditions. These are namely Alzheimer’s, Parkinson’s, and Huntington’s diseases [14,22], and it has exhibited protective properties in multiple investigative models of neurodegenerative conditions [23,24].

## 4. Potential Mechanisms through Which Nicotinamide May Exert Its Effects in Glaucoma Management

Although raised IOP appears to be the primary cause of glaucoma, which might hinder axonal transmission among the neuronal tissue that make up the second cranial nerve [25,26], other variables might also exacerbate RGC mortality, either independently or in concert with increased IOP [27]. 

Impaired blood flow autoregulation leading to local hypoxia [1], glutamatergic system overstimulation [28,29], abnormal immunological responses [30], oxidative stress [31,32], and mitochondrial dysfunction [33,34,35,36] are some of the causes that may be linked to RGC depletion. 

Among these variables, oxidative damage and mitochondrial dysfunction are strong reasons to investigate the possible utility of NAM and its riboside in treating glaucoma, considering their anti-oxidative qualities and capacity to modulate mitochondrial activity [9,16,37]. Zeng has explored the possible impacts and processes of NR on a fibrosis and oxidative stress model using cells from the human trabecular meshwork (HTM). The study found that NR pretreatment boosted the survival and proliferation of HTM cells, protecting them against oxidative stress and fibrosis [38].

Camalleri et al. observed that dietary supplementation with antioxidant compounds, including a combination of forskolin, homotaurine, and spearmint extract, with selected B vitamins, resulted in the restoration of impaired retinal parameters assessed through electrophysiological examinations. Consequently, the authors suggested possible neuroprotection alternatives independent of IOP, proposing that dietary supplementation might attenuate inflammatory processes initiated by glial cell activation, thereby safeguarding RGCs [39]. 

In glaucoma, the elevated mutation rate observed in mitochondrial DNA has proven the presence of a mitochondrial dysfunction [40,41]. Furthermore, the activity of the mitochondrial respiratory complex I seems to be reduced by 18% in the lymphoblasts of primary open-angle glaucoma sufferers when compared to a non-POAG group (*p* = 0.032), resulting in comparable reductions in ATP production in both groups [42]. The redox imbalance in glaucoma is worsened due to the astrocyte and RGC mitochondrial malfunction in [43], that hampers the sustenance of the increased energy request due to metabolic stress in cases of higher IOP [1].

Moreover, there is an age-related increase in NAD consumption, resulting in diminished NAD availability across various tissues. Notably, this reduction in NAD levels is particularly pronounced in individuals with glaucoma [14,44,45].

A reduction in NAD levels increases the likelihood of mitochondrial malfunction, making RGCs more vulnerable to extrinsic influences such a high IOP [14,45,46,47]. Notably, individuals with POAG had a 30% lower plasma level of NAM, a precursor of NAD, compared to age- and gender-matched controls [48,49]. However, systemic NR administration causes a considerable rise in NAD bioavailability in numerous tissues, inclusive of components of the central nervous system [24,50]. It, therefore, makes sense that dietary supplementation with either NAM or NR, aiming at increasing the NAD bioavailability, might offer a viable therapeutic strategy in the treatment of glaucoma [33,51].

## 5. Studies on the Use of NAM and NR in Glaucoma

There have been many animal studies looking at the potential of NAM as a neuroprotective agent in glaucoma. Williams et al. compared mitochondrial abnormalities and lower NAD+ concentrations in DBA/2J mice, a glaucoma-like rodent model, to controls [52]. They next showed that the supplementation of exogenous nicotinamide (at a level comparable to 2.5 g daily in a 60 kg human) may restore the function of RGCs in elderly mice prone to glaucoma mice, resulting in a tenfold reduction in the chance of developing glaucoma [47,52].

Chou et al. studied D2 mice utilizing flickering-light-pattern electroretinography (F-PERG) to determine the effect of nicotinamide supplementation (equivalent dosage of 2 g per day) on RGC function. The adaption of F-PERG declined with age and was significantly poorer in untreated D2 mice (*p* < 0.01). Furthermore, they found that, utilizing immunohistochemical methods, the treated group had a higher RGC density than the control group [53].

Zhang et al. administered mice with nicotinamide riboside or phosphate-buffered saline (PBS) daily for five days to see how NR treatment affected NAD levels in RGCs. In comparison to the PBS-injected control group, NR treatment almost quadrupled NAD levels in the retina (*p* < 0.01) [54].

Tribble et al. conducted another investigation on mice with ocular hypertension and found that nicotinamide therapy was effective. They demonstrated that nicotinamide may increase mitochondrial activity and have a neuroprotective impact on defective RGCs. Using a retinal explant model, they also investigated the protective benefits of nicotinamide (NAM) against an acute, axon-specific damage. Retinas dissected and cultivated in solutions diluted with two doses (100 mM or 500 mM) of NAD showed the reduced loss of retinal RGCs and less shrinkage of cell nuclei as compared to retinal explants cultured ex vivo without NAM supplementation [9].

Yu et al. examined RGC alterations in rat models of chronic ocular hypertension. After providing NADPH and N-acetylcysteine (NAC), they detected decreased glutathione (GSH) production, apoptosis, axonal damage, and peroxidation in RGCs. Furthermore, electrophysiological function increased while Müller cell gliosis was decreased [55].

Boodram evaluated the impact of dietary nicotinamide supplementation on axonal microtubule degradation, which seems to play an important part in the development of glaucoma. They discovered that administering 550 mg per day in a mouse model of glaucoma protected the volume of retinal nerve fibers [56]. Kim et al. assessed a direct administration approach using NAM extracellular vesicles (EVs) in mice glaucoma models. A quantitative study of dendritic integrity demonstrated NAM’s ability to protect RGCs [57].

## 6. Clinical Evidence on the Use of NAM or NR in Glaucoma

Hui et al. carried out a randomized, double-blind, case–control investigative study on 57 POAG patients, separated into two categories. One set of mice received NAM at 1.5 g/day for 6 weeks, then half the starting dose for the remaining 6 weeks, whereas another set group received a placebo for 3 months. After 12 weeks, the groups changed treatments. Assessments, including comprehensive eye examinations, visual field tests, and electroretinography, were performed at the beginning and every six weeks [8].

Out of the 49 participants who took part in the study, those who received NAM showed improved electroretinographic characteristics associated with the inner retina (*p* = 0.02). Slight changes in automated perimetry were observed. In the group that took 3 g/day of NAM, there was a greater improvement in the mean deviation (MD) of ≥1 dB from the baseline compared to the placebo group (27% vs. 16%, *p* = 0.02). Patients who took a high dose of NAM had fewer decreases in MD of ≥1 dB when compared to the placebo group (4% vs. 12%, *p* = 0.02). However, after 12 weeks, no significant difference was observed in the average MD between the placebo and treatment groups (*p* = 0.53). NAM supplementation did not have any effect on the intraocular pressure (IOP), mean arterial pressure, visual acuity, or retinal nerve fiber layer thickness. However, this research was limited by an inadequate follow-up period, different forms of glaucoma in the participants, and incomplete ocular pressure data during the study [8].

Moraes et al. conducted a double-masked experiment on POAG, assessing the effects of NAM- and pyruvate (PYR)-fortified diets. Thirty-two participants with moderate POAG were placed into two groups: one group received up to 3000 mg/day of dietary NAM and PYR for 6 weeks, while the second group received a placebo for the same time period. Participants were subjected to four visual field tests spanning two weeks at the beginning and conclusion of the research, with two extra tests administered four weeks in and one week after supplementation finished. 

The treatment group had a significantly average improvement on the total deviation plot (*p* = 0.005), and a threefold higher probability of improving the thresholds of the tested points (*p* = 0.02), notwithstanding the initial sensitivity, and higher rates of improvements in the pattern standard deviation (PSD). However, there were no significant changes in the visual field indices, mean deviation, ocular coherence tomography, nor MoCA scores.

The findings revealed that the locations exhibiting improvement initially had an intermediate sensitivity, indicating that supplementing may be improving the function of the defective RGCs. Despite the apparent functional gains, the study’s brief length precludes the extrapolation to long-term effects, and no information on IOP behavior in either group was found [58].

Leung et al. performed a randomized clinical study at the Chinese University of Hong Kong to assess the outcome of 300 mg of NR augmentation on 125 POAG patients who were randomly allocated to receive either NR or a placebo over 24 months. Ocular examinations of the retinal nerve fiber layer (RNFL) and visual field (VF) tests are planned at the beginning of the research, after one month, four months, and then every two months until the 24-month period is completed. The major aim is to establish if NR may delay the pace of RNFL thinning over 24 months by evaluating the VF progression and OCT parameters [59].

Thus far, four important ongoing clinical studies deserve to be noted.

Garthway-Heath et al. are carrying out a random-sampled, placebo-controlled, multicenter, phase 3 trial at the University College of London, to measure the reduction in average visual field sensitivity over 27 months in POAG sufferers administered an increasing dose of NAM (from 1.5 g per day for 6 weeks, then rising to 3.0 g daily) compared to those receiving placebo tablets, as well as to assess the safety of NAM and its effects on mitochondrial activity (NCT05405868) [60].

Another prospective, randomized, placebo-controlled, double-masked trial led by Jennie Nyman at Umea University, Sweden and the Australian Centre for Eye Research (NCT05275738) aims to assess the VF progression indices, with the participants to be administered 3.0 g/day of NAM or a placebo over a two-year period [61].

Furthermore, Kolomeyer is conducting another work of clinical research that intends to assess the changes in vision and visual function after six months of therapy. POAG subjects are being administered GlaucoCetin containing NAM, while a control group receives a placebo. The trial will also evaluate improvements in quality of life, electrophysiological responsiveness, and contrast sensitivity (NCT04784234 [62]).

Finally, Columbia University is conducting an interventional, randomized clinical trial to investigate changes in the central visual field, RNFL, and RGC layer using optical coherence tomography in 188 OAG patients that will be taking pyruvate and nicotinamide versus placebo for a total of 20 months (NCT05695027) [63].

## 7. Safety Profile

Nicotinamide is considered a dietary supplement rather than a drug formulation. The oral administration of this substance results in efficient absorption and distribution throughout the body’s tissues. It undergoes hepatic metabolism and renal excretion. The recommended daily nicotinamide intake is about 15 mg, and the occurrence of negative effects is uncommon, even at the high levels used in pharmacological treatments. Even when these negative effects occur, they are generally mild and may include gastrointestinal discomfort [58], headache, nausea, tinnitus [8], and vertigo. These complaints were reported to resolve spontaneously after stopping NAM administration [64]. Only one serious adverse event of drug-induced liver injury (DILI) has been reported related to the use of NAM. A 73-year-old woman who was participating in the NCT05695027 trial, dosing over the first 3 weeks, with 1 g/day of nicotinamide and 3 g/day of calcium pyruvate in the first week, 2 g/day of each component in the second week, and 3 g/day for each element in the third week, experienced severe acute transaminitis within 5 weeks of treatment. However, the DILI appeared to be idiosyncratic and not related to the pharmacological properties of the drug [65]. Concerns have been raised about its safety in long-term use due to a hypothesis of increased cell proliferation and tumorigenesis following the erased intracellular NAD. Maric et al. has reported a higher prevalence of neoplastic growths in the mammary glands of murine models treated with NR, but the study lacked statistical analysis, and the dose used (400 mg/kg) was extremely high compared to typical human doses [66]. 

On the opposite hand, NAD may actually prevent cancer, as it is a substrate for the DNA-repair enzyme PARP-1, which prevents cancer-promoting mutations [67]. Additionally, NAD supplementation has been shown to improve the effectiveness of tumor immunotherapy [68]. A human study found that nicotinamide boosted the metabolism and survival of natural killer cells, aiding in remission in non-Hodgkin lymphoma patients [69]. Moreover, no increased tumor incidence has been reported among subjects taking high doses of nicotinamide for dermatological disorders or cancer prevention, nor in diabetic patients on high doses for prolonged periods [70,71]. Glaucoma management needs to be personalized and tailored to the severity of the disease and the individual needs of the patients to ensure a proper quality of life. It is imperative that patients undergo pertinent testing, examinations, differential diagnosis, and treatment to provide the best clinical outcomes and treatments for each patient [72,73,74].

While generally well-tolerated, nicotinamide can pose risks for certain individuals. People with pre-existing liver disease or a history of jaundice should exercise caution due to the potential worsening of liver function. Similarly, those with diabetes may experience blood sugar fluctuations, so monitoring blood sugar levels is crucial [75]. Nicotinamide might also interact with medications that affect blood clotting or how the body breaks down certain drugs, so it is important to consult a doctor before taking it alongside other medications.

## 8. NAD Fortification and Supplementation in the Body

Multiple compounds have been reported to increase NAD levels in the body. Van der Muelen et al. [76] carried out a study on bifidobacterial metabolism. They reported that sugar consumption and subsequent succinic acid production resulted in the production of NAD+. Apigenin has also been shown to upregulate NAD dehydrogenase quinone [77], and increase the activation factor of NAD [77]. Resveratrol is another compound, and it has been reported to show synergistic activities with other compounds, promoting the production of NAD+, thereby prolonging good health [78]. Nicotinamide is, however, a drug of choice because of its ability to augment the activity of other NAD+-promoting compounds [77]. Glaucomatous damage results from increased pressure intraocularly and can begin at any point in the disease progression [79]. Hence, NAM supplementation/intake is advised at all times.

## 9. Review Summary

A summary of the sampled publications used in this paper is shown in Table 1 below.

## 10. Limitations to This Study

Nicotinamide is a novel therapy for glaucoma management. Hence, the literature on its efficacy and comparative use is relatively scarce. The authors suggest that this review should spur further research on this nutrient. Moreover, while this review correctly describes the usefulness of Nicotinamide as a drug, as shown in its safety profile above, there still needs to be concern about the clinically safe dosing and treatment regimen. 

Another limitation encountered in the course of this review was the relative paucity of data evidenced by the low number of records returned by the search criteria. The authors attempted to widen the search net but the records returned were not relevant to this study.

## 11. Conclusions

The quest for new treatments to enhance glaucoma management is ongoing. Studies have indicated that NAM and nicotinamide riboside (NR) support mitochondrial health and may shield RGCs from stressors like increased IOP. These protective effects have been confirmed in various experimental models of axonal injury. Short-term NAM supplementation has improved retinal function and visual field sensitivity in clinical settings. NR is currently being evaluated in glaucoma patients, though no results have been published yet. While these compounds generally have a good safety record, their long-term effects are still unclear. Additionally, there is no definitive pharmacokinetic study that establishes the bioavailability of NMN when administered orally or topically. As a result, the bioavailability of NMN remains uncertain and could potentially limit its effectiveness and use.

## Figures and Tables

**Table 1:** Summary of sampled studies.

Author	Type of Study	Study Sample	Methods	Conclusion
Hui et al. [8]	In vivo	57 glaucoma patients	Change in inner retinal function	Nicotinamide supplementation can improve inner retinal function.
Tribble et al. [9]	In vivo	Murine models	Optical coherence tomography of the outer RGC layer	A nicotinamide-enriched diet significantly reduced RGC loss compared to normal diet.
Chou et al. [53]	In vivo	10 DBA/2J mice	Electroretinogram (PERG) of RGC function	NAM-fed D2 showed increased RGC density (2.4×), and larger RGC soma size (2×) when compared to controls
Zhang et al. [54]	In vivo	Murine models	PERG of RGC function	Mice treated with NAM precursors showed significantly preserved RGC function.
Nzoughet et al. [48]	In vitro	34 POAG patients	Liquid chromatography and mass spectrometry was used to assess NAM levels in blood plasma compared to controls.	Plasma NAM levels were significantly lower in the glaucoma cohort when compared with controls.
Williams et al. [47]	In vitro	Murine models	Metabolites of age- and glaucoma-compromised RGCs were studied (outer retina).	NAD and NAD+ levels were found to be reduced, suggesting they play a role in keeping RGCs healthy.
Zeng et al. [38]	In vitro	Human trabecular meshwork cells (HTM)	Hoechst staining and MTT assays were used to assess HTM viability.	NAM had a protective effect on oxidative stress, prolonging HTM viability.
Sasaki et al. [37]	In vitro	Mouse models	Axonal degeneration in extracted dorsal root ganglia (DRG) from rat embryos	NAM synthesized from different precursors slowed the rate of axonal degeneration.
Tribble et al. [9]	In vitro	Murine models	Observed nuclear shrinkage in cultured RGC axons	NAM fortification resulted in less RGC axon loss and shrinkage.

## Data Availability

Data sharing is not applicable.
